# Van der Waals Gap‐Confined Molecular Engineering of MoS_2_ for Selectivity‐Enhanced and Humidity‐Tolerant Gas Sensing

**DOI:** 10.1002/advs.78072

**Published:** 2026-10-08

**Authors:** Jae Hyung Shim, Gwang Su Kim, Tae Hyung Lee, Jaeho Lee, Su‐Yeon Joung, Jung Hoon Kang, Jin Seok Kim, Jung‐Won An, Chong‐Yun Kang, Ho Won Jang, Jungwon Park, Ji‐Soo Jang, Chul‐Ho Lee

**Affiliations:** ^1^ Department of Electrical and Computer Engineering Seoul National University Seoul Republic of Korea; ^2^ Inter‐University Semiconductor Research Center (ISRC) Seoul National University Seoul Republic of Korea; ^3^ Seoul National University Energy Initiative (SNUEI) Seoul National University Seoul Republic of Korea; ^4^ Electronic and Hybrid Materials Research Center Korea Institute of Science and Technology (KIST) Seoul Republic of Korea; ^5^ Department of Materials Science and Engineering Seoul National University Seoul Republic of Korea; ^6^ Department of Chemical and Biological Engineering Seoul National University Seoul Republic of Korea; ^7^ Advanced Institutes of Convergence Technology Seoul National University Gyeonggi‐do Republic of Korea; ^8^ Center for Nanoparticle Research Institute for Basic Science (IBS) Seoul Republic of Korea; ^9^ Institute of Engineering Research College of Engineering Seoul National University Seoul Republic of Korea; ^10^ SKKU Advanced Institute of Nanotechnology and Department of Nanoengineering Sungkyunkwan University Suwon Republic of Korea

**Keywords:** defect engineering, humidity‐tolerant gas sensing, room‐temperature gas sensor, two‐dimensional materials, van der waals gap engineering

## Abstract

Controlling interactions between sensor surfaces and target molecules is central to achieving high selectivity and stability in chemical sensing. However, in two‐dimensional (2D) material‐based sensors, unintended adsorption, particularly of water molecules, often disrupts these interactions, degrading selectivity and long‐term stability. Here, we exploit molecular confinement within van der Waals (vdW) gaps of MoS_2_ to engineer sensor–analyte interactions. Ethanol (EtOH) confinement induces electron doping and hydrophobicity, whereas water confinement produces negligible doping and hydrophilicity. This combined modulation suppresses H_2_O adsorption while promoting selective NO_2_ adsorption, enabling the EtOH‐confined sensor to detect NO_2_ from 200 ppb to 5 ppm, with a maximum response of 48% at 60% relative humidity and a detection limit of 36 ppb. Under highly humid conditions, it exhibits a sevenfold higher NO_2_ response and retains 95% of its initial response after 5 weeks, compared with 44% for H_2_O‐confined films. First‐principles calculations reveal that molecular confinement regulates sulfur‐vacancy (V_s_) configurations: EtOH favors isolated and lined V_s_, which promote NO_2_ adsorption while suppressing H_2_O binding, whereas H_2_O stabilizes clustered V_s_, which increase H_2_O affinity. This vdW‐gap confinement provides a design strategy to tune surface chemistry, electronic structure, and defect configurations for selective, humidity‐tolerant, and reliable 2D chemical sensors.

## Graphical abstract

Molecular confinement within van der Waals gaps of MoS_2_ is established as a powerful design strategy for selective and stable chemical sensing. Ethanol‐confined films simultaneously tune electronic doping, surface wettability, and sulfur vacancy configurations, suppressing water interference while enhancing NO_2_ adsorption. The resulting sensors show a sevenfold response increase and 95% response retention after 5 weeks under highly humid conditions.

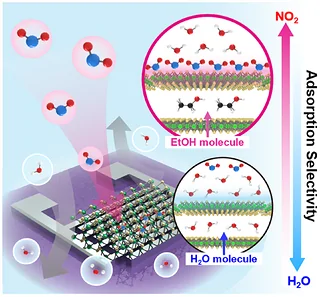

## 1 Introduction

Surface interactions between sensing materials and target molecules play a central role in determining the performance of chemical sensors [1, 2, 3]. In particular, the efficiency of selective gas adsorption and the subsequent charge transfer processes at the material surface critically governs sensing responses. However, real‐world operating environments introduce various complexities, including competitive adsorption, non‐specific molecular interactions, and environmental fluctuations [4, 5, 6, 7]. Among these factors, humidity represents one of the most critical challenges, as water molecules can severely compromise selectivity and long‐term operational stability [8, 9]. High humidity can induce surface screening effects, perturb charge redistribution at the sensing interface, weaken selective gas adsorption, and accelerate material degradation over time [10, 11, 12]. These issues become particularly pronounced in nanomaterial‐based sensors, where the large surface‐to‐volume ratio enhances response but simultaneously amplifies susceptibility to environmental interference. Consequently, developing material systems and interfaces that maintain selective surface interactions while resisting moisture interference is essential for reliable and long‐term chemical sensing operation.

Two‐dimensional (2D) materials have attracted significant attention as versatile platforms for adsorption‐driven sensing applications, owing to their unique physical and chemical properties [13, 14, 15, 16]. Their atomically thin structure provides a high surface‐to‐volume ratio, enabling efficient interactions with gas molecules, while their electronic structure can be readily tuned to modulate surface reactivity and charge transfer dynamics. These characteristics make 2D materials highly promising candidates for gas sensing technologies [17, 18, 19].

However, the fully exposed nature of their atomically thin surfaces makes them particularly vulnerable to environmental perturbations, among which humidity is one of the most critical factors affecting sensing performance and reliability. Under humid conditions, water molecules readily adsorb onto the surface, frequently outcompeting target gas species for active sites. This competitive adsorption disrupts selective sensing and alters the local charge environment, thereby hindering effective charge transfer between the sensing material and the adsorbed gas molecules [20, 21]. Over time, such processes can lead to severe degradation in sensing characteristics, including reduced response, baseline instability, and slower recovery dynamics [10, 12, 22, 23]. Moreover, prolonged exposure to moisture can accelerate structural and chemical degradation of the sensing material itself. For example, hydroxylation or partial oxidation may occur in the presence of reactive gases or elevated temperature, leading to irreversible changes in the electronic structure of the sensing material [8, 24]. These effects significantly limit the long‐term reliability of 2D‐material‐based gas sensors, particularly under realistic operating environments where humidity levels are high and fluctuate significantly. Therefore, overcoming humidity‐induced degradation remains a critical challenge in the practical deployment of 2D gas sensing platforms.

To address these challenges, various engineering strategies have been explored. Nanostructuring and heterostructure integration have been widely employed to enhance gas‐surface interactions by increasing the active surface area or introducing built‐in electric fields that facilitate charge separation and transport [17, 25, 26, 27]. These approaches have been extensively adopted in recent room‐temperature NO_2_ sensors based on various nanostructured and heterostructured materials [28, 29, 30, 31]. While they effectively improve sensitivity and response dynamics, they are not inherently designed for adsorption selectivity or humidity tolerance. Additional approaches, including encapsulation, surface functionalization, and controlled oxidation, have been developed to limit water adsorption or modify surface hydrophobicity [10, 24, 32]. In some cases, external stimuli such as ultraviolet illumination or elevated temperatures are used to desorb moisture and recover sensing responses [33, 34]. However, these strategies often introduce additional device complexity, increase energy consumption, or compromise gas selectivity. More importantly, they primarily function as external or post‐treatment solutions rather than directly modifying the intrinsic surface environment that governs competitive adsorption and charge transfer processes. Moreover, most existing strategies have been developed and optimized under dry or low‐humidity conditions, where high initial response is often achieved at the expense of long‐term operational stability in humid environments. These limitations highlight the need for a fundamentally different strategy that directly engineers the surface interaction landscape, rather than merely mitigating the effects of humidity. Such a strategy should intrinsically regulate competitive adsorption at the sensing interface, thereby enabling selective and stable gas sensing under humid conditions.

In this study, we introduce a van der Waals (vdW) gap molecular confinement strategy in MoS_2_ to regulate surface interactions for highly selective and stable gas sensing under humid conditions. By confining specific molecules within the interlayer vdW gaps, this approach simultaneously modulates the electronic structure and the spatial configuration of sulfur vacancies (V_s_), thereby tailoring the surface reactivity of MoS_2_ toward NO_2_. Although sulfur vacancies in MoS_2_ are widely recognized as active sites for NO_2_ adsorption, their effectiveness is often limited by competitive adsorption and environmental interference, particularly under humid conditions. We demonstrate that the spatial configuration of sulfur vacancies, rather than their density, governs selective NO_2_ sensing under humid conditions. Ethanol‐ and water‐confined MoS_2_ possess comparable vacancy densities yet adopt distinct vacancy configurations, which we identify as the origin of their markedly different sensing behavior. Experimentally, ethanol‐confined MoS_2_ (EtOH‐MoS_2_) exhibits a sevenfold higher NO_2_ response than water‐confined MoS_2_ (H_2_O‐MoS_2_) under humid conditions, and retains 95% of its initial response after 5 weeks at 60% RH, whereas H_2_O‐MoS_2_ retains only 44%. By simultaneously achieving high sensing performance under humid conditions and long‐term operational stability, this work addresses a longstanding challenge that has remained largely unresolved by conventional response‐oriented strategies.

## 2 Results and Discussion

Figure 1a schematically illustrates the concept of vdW‐gap‐confined molecular engineering and how adsorption selectivity at the surface of 2D materials is governed by the type of molecules confined within the vdW gap. Under humid conditions, abundant water molecules can interfere with selective NO_2_ adsorption and influence surface stability. In this framework, MoS_2_ films with ethanol confinement preferentially adsorb NO_2_ while suppressing H_2_O adsorption, whereas water‐confined MoS_2_ films show dominant H_2_O adsorption with reduced selectivity for NO_2_. This schematic highlights how molecules confined within vdW gaps regulate competitive adsorption behavior, leading to distinct adsorption selectivity under humid environments.

**FIGURE 1:**
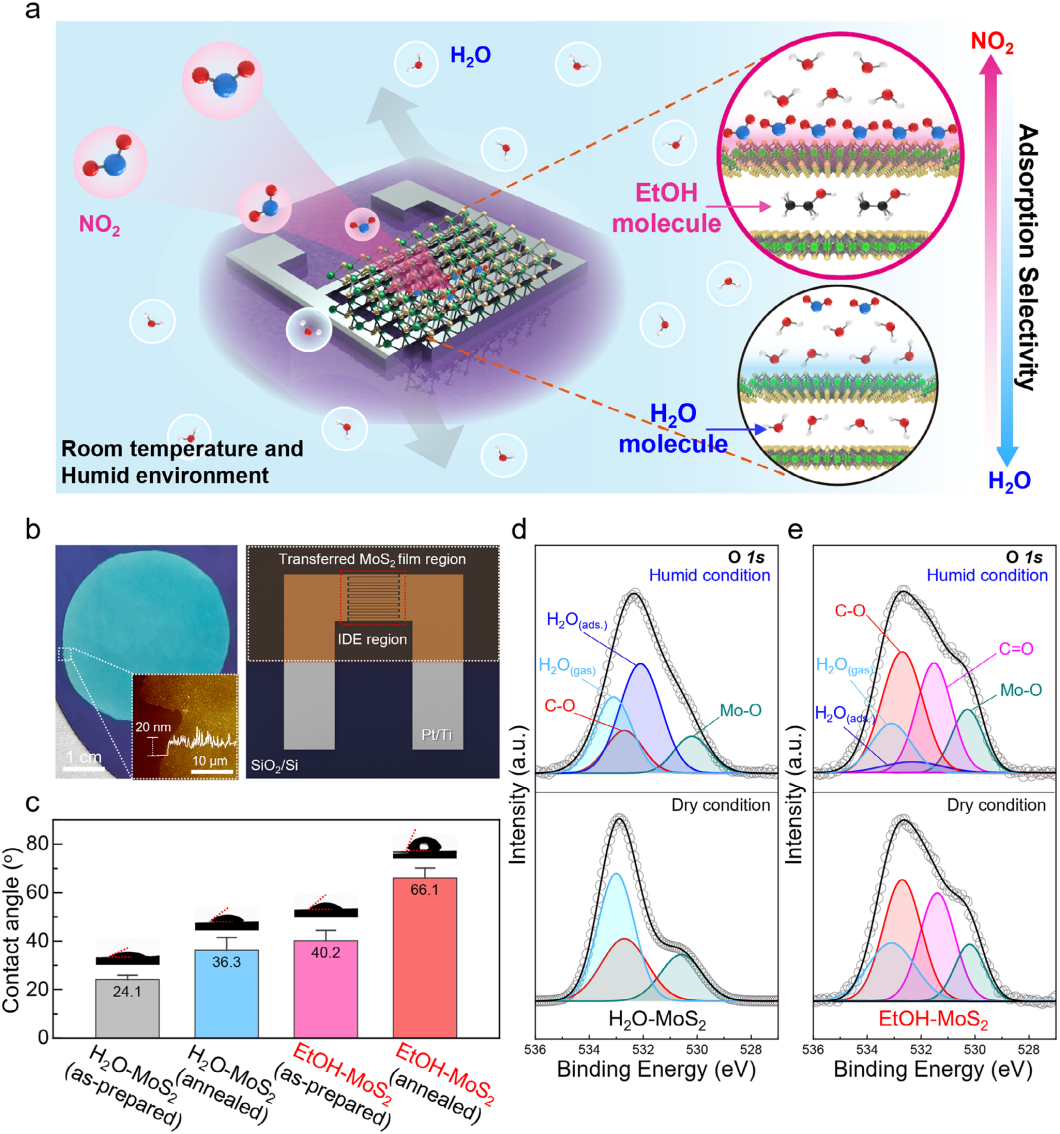
Selective gas adsorption and humidity‐tolerant behavior enabled by vdW‐gap molecular confinement in MoS_2_ films. (a) Schematic illustration of the proposed mechanism, where specific molecules confined within the vdW gaps modulate the surface chemistry of MoS_2_, promoting NO_2_ adsorption while suppressing H_2_O interference. (b) Optical images of the as‐prepared MoS_2_ films transferred onto a SiO_2_/Si substrate (left) and the IDE‐patterned gas sensor architecture (right). (c) Water contact angle measurements of H_2_O‐ and EtOH‐confined MoS_2_ films before and after annealing. (d) O 1s XPS spectra of annealed H_2_O‐MoS_2_, and (e) EtOH‐MoS_2_ films, before and after prolonged exposure to high humidity, highlighting their distinct water adsorption behavior.

To experimentally realize this concept, molecule‐confined MoS_2_ films were prepared via chemical exfoliation followed by vacuum‐assisted assembly (Figure S1). Bulk 2H‐phase MoS_2_ was first chemically exfoliated through lithium intercalation to produce 1T‐phase MoS_2_, which readily dispersed into monolayer nanosheets in water. For the formation of molecule‐confined MoS_2_ films, the aqueous dispersion of exfoliated nanosheets was mixed with molecule‐rich solvents such as ethanol and assembled into freestanding films via vacuum filtration. During this process, excess solvent molecules attach to defects and become confined within the interlayer vdW gaps between stacked MoS_2_ nanosheets. As a result, the solvent used during filtration determines the molecules confined within the MoS_2_ interlayers, thereby modulating the interlayer environment and surface properties of the resulting films.

The optical image in Figure 1b shows centimeter‐scale EtOH‐MoS_2_ films uniformly transferred onto a SiO_2_/Si substrate, with a thickness of approximately 20 nm as confirmed by atomic force microscopy (AFM, Figure S2). Interdigitated electrodes (IDEs) were subsequently fabricated on top of the film, enabling a gas sensor configuration, in which the MoS_2_ film serves as the active channel.

Figure 1c compares the surface wettability depending on the type of confined molecule and subsequent thermal treatment. The as‐prepared H_2_O‐MoS_2_ film exhibits a relatively hydrophilic surface with a contact angle of 24.1°, which moderately increases to 36.3° after annealing at 200°C. In contrast, the EtOH‐MoS_2_ film shows a pronounced increase in hydrophobicity upon the same thermal treatment, with its contact angle rising from 40.2° to 66.1°. The full temperature dependence is shown in Figure S3, where both films exhibit similar contact angles of 59.8° and 58.2°, respectively, after annealing at 300°C. These results indicate that vdW‐gap‐confined molecules strongly influence surface wettability, particularly after thermal treatment.

To investigate the water adsorption behavior under high‐humidity conditions, surface chemical changes were monitored over 24 h using x‐ray photoelectron spectroscopy (XPS). The O 1s spectra reveal contributions from both surface‐adsorbed water molecules and gas‐phase water species. For the annealed H_2_O‐MoS_2_ films, a distinct peak at 533.1 eV corresponds to gas‐phase water molecules, and prolonged exposure to humid air results in a pronounced increase in the adsorbed H_2_O peak near 532.2 eV, indicating substantial water adsorption on the surface [35, 36] (Figure 1d).

In contrast, the annealed EtOH‐MoS_2_ films exhibit minimal changes in water‐related signals under the same conditions (Figure 1e). Under dry conditions, characteristic peaks corresponding to C─O (532.8 eV), C═O (531.2 eV), and Mo─O (530.3 eV) are observed [37, 38], and even after extended humidity exposure, the H_2_O_(ads.)_ peak remains weak. This behavior is attributed to enhanced surface hydrophobicity induced by ethanol confinement within the vdW gaps.

To understand the origin of the distinct surface wettability and adsorption behavior, we investigate how molecular confinement within vdW gaps modulates the interlayer structure and electronic properties of MoS_2_. During chemical synthesis, a relatively high density of sulfur vacancies forms on the MoS_2_ surface, providing active sites for interactions with small molecules [38, 39, 40], as illustrated in Figure 2a. These interactions lead to interlayer expansion and facilitate charge transfer between confined molecules and the MoS_2_ lattice.

**FIGURE 2:**
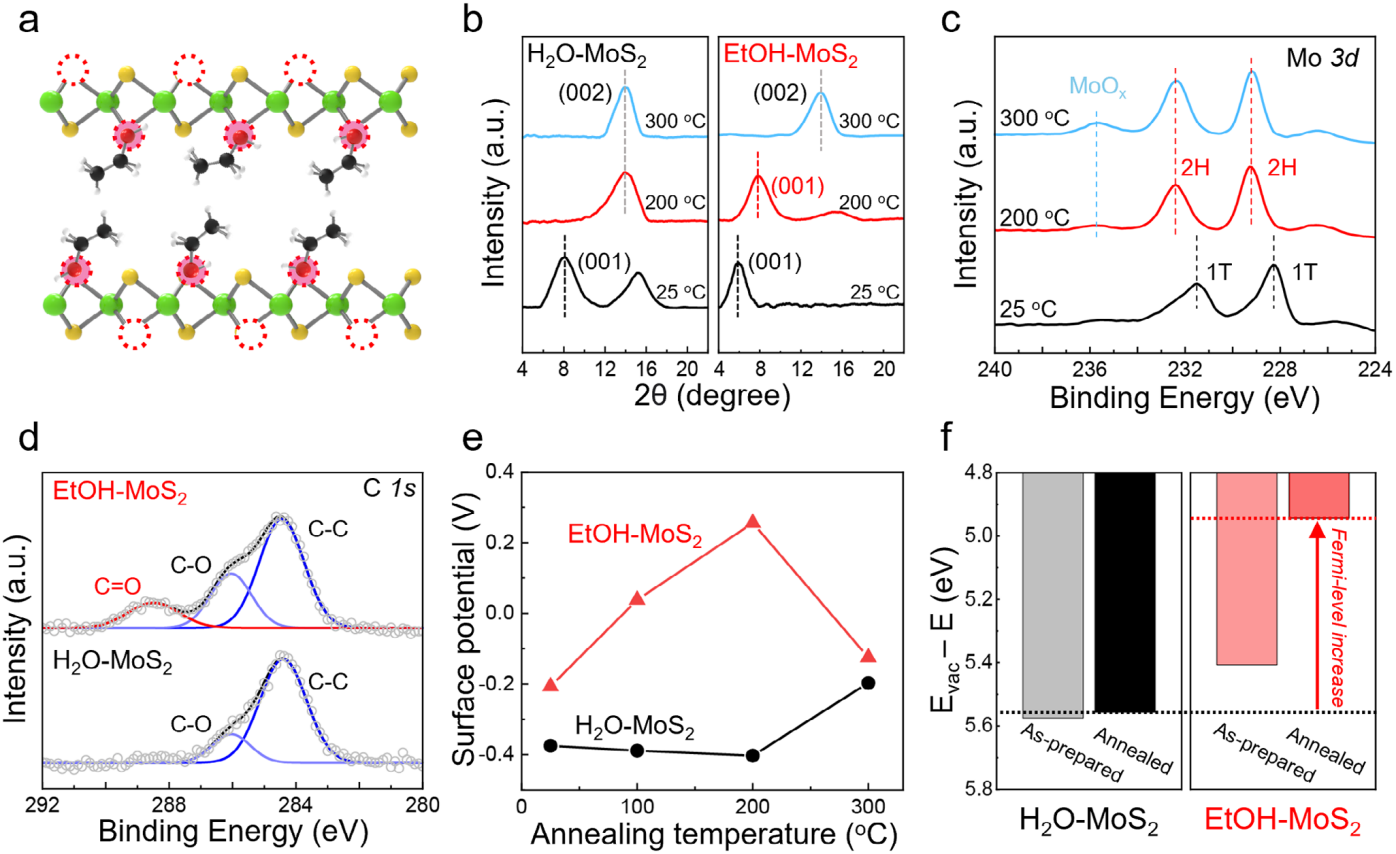
Structural, chemical, and electronic modulation in MoS_2_ films induced by vdW‐gap molecular confinement. (a) Schematic of the molecule‐confined MoS_2_ structure, illustrating the insertion of molecules within the vdW gap. (b) X‐ray diffraction patterns of H_2_O‐MoS_2_ and EtOH‐MoS_2_ films under different annealing temperatures (RT, 200°C, and 300°C), showing the molecule‐dependent evolution of interlayer spacing. (c) Temperature‐dependent phase transition of EtOH‐MoS_2_ from 1T to 2H, identified by Mo 3d XPS spectra. (d) C 1s XPS spectra revealing the presence and chemical state of confined molecules. (e) Surface potential variation measured by KPFM as a function of annealing temperature, showing thermally activated electronic modulation. (f) Work function changes of H_2_O‐ and EtOH‐ MoS_2_ films after annealing at 200°C, demonstrating an upward Fermi‐level shift and the associated electron doping effect.

To elucidate the structural evolution induced by confined molecules, interlayer spacing was analyzed using x‐ray diffraction (XRD). The patterns in Figure 2b and Figure S4 reveal that the vdW gap strongly depends on the confined molecular species. For H_2_O‐MoS_2_, a (001) diffraction peak at room temperature (RT) corresponds to an extended interlayer spacing of 1.1 nm, indicating water intercalation [41]. Upon annealing at 200°C and 300°C, this peak disappears and is replaced by the (002) peak of the 2H phase, with the spacing contracting to 0.63 nm, reflecting complete water desorption and structural recovery to the thermodynamically stable configuration.

In contrast, EtOH‐MoS_2_ exhibits a larger interlayer spacing of 1.4 nm, which decreases only slightly to 1.1 nm after annealing at 200°C, indicating stable retention of ethanol within the interlayer space. Only at 300°C does the structure revert to the 2H phase with a spacing of 0.63 nm, confirming complete ethanol removal. These results demonstrate that ethanol exhibits stronger confinement and higher thermal stability within vdW gaps than water, highlighting the critical role of molecular species in governing the structural evolution of layered MoS_2_ systems. The confinement‐induced change in interlayer spacing was further verified by cross‐sectional HRTEM (Figure S4c). After annealing at 200°C, EtOH‐MoS_2_ exhibits an interlayer lattice spacing of 1.0 nm, whereas H_2_O‐MoS_2_ shows 0.62 nm, in close agreement with the corresponding XRD values (1.1 and 0.63 nm, respectively). These direct observations independently confirm the retention of the expanded vdW gap in the ethanol‐confined film.

The phase evolution was further examined by XPS (Figure 2c and Figure S5) and Raman spectroscopy (Figure S6). At RT, EtOH‐MoS_2_ predominantly exhibits the metallic 1T phase, with Mo 3d_5/2_ and Mo 3d_3/2_ peaks located at 228.2 and 231.4 eV, respectively, along with a minor 2H component (Mo 3d_5/2_ and Mo 3d_3/2_ peaks at 229.2 and 232.3 eV). Upon annealing at 200°C, the spectra indicate a transition toward the 2H phase, accompanied by a weak MoO_x_ feature that grows markedly by 300°C, indicating progressive partial oxidation. Quantitative deconvolution of the Mo 3d spectra shows nearly identical fitted 2H spectral‐area fractions for EtOH‐MoS_2_ and H_2_O‐MoS_2_ at 200°C (81.0% and 81.5%), with only a 2.2 percentage‐point difference in the fitted Mo^6+^ spectral‐area fractions associated with MoO_x_ (13.5% and 11.3%, respectively; Figure S7). The phase transition, the loss of confined species, and partial oxidation occur over similar temperature ranges, and each contributes to changes in surface adsorption and charge transfer characteristics. Because these processes occur simultaneously, their individual contributions cannot be completely decoupled. Therefore, the effect of molecular confinement is evaluated by directly comparing EtOH‐MoS_2_ and H_2_O‐MoS_2_ under identical thermal treatment conditions. Similar behaviors are observed in H_2_O‐MoS_2_, indicating that the confined molecules do not significantly alter the intrinsic 1T‐to‐2H phase transition pathway.

To identify the chemical nature of confined species, C 1s XPS spectra were analyzed (Figure 2d and Figure S8). A distinct peak at 288.3 eV corresponding to C═O bonding is observed in EtOH‐MoS_2_, indicating the formation of partially oxidized ethanol derivatives. The absence of this peak in H_2_O‐MoS_2_ confirms that ethanol molecules not only remain confined but also undergo partial oxidation. Depth‐profile XPS after annealing at 200°C further shows a higher inner‐film C 1s/Mo 3d ratio for EtOH‐MoS_2_ than for H_2_O‐MoS_2_ (0.108 ± 0.029 and 0.050 ± 0.017, respectively; Figure S9b). The averaged inner‐film C 1s spectrum of EtOH‐MoS_2_ also contains a component near 288.6 eV in the carboxylate binding‐energy range, whereas the intensity in the corresponding region of H_2_O‐MoS_2_ is too low for reliable component fitting (Figure S9c–f). Together with the retained interlayer expansion (Figure 2b and Figure S4), these results support the presence of ethanol‐derived carboxylate‐like species within the expanded vdW gaps of EtOH‐MoS_2_ rather than exclusively as surface‐bound carbon species. This result directly reveals the origin of electron doping induced by ethanol confinement. Together, these results establish a direct link between molecular confinement, chemical transformation, and electronic doping in MoS_2_.

To probe the resulting electronic modulation, Kelvin Probe Force Microscopy (KPFM) was employed (Figure 2e and Figure S10) [42]. The EtOH‐MoS_2_ films exhibit uniformly higher in surface potential from −207 mV to +256 mV upon annealing at 200°C, followed by a decrease to −125 mV at 300°C. In contrast, the H_2_O‐MoS_2_ films show minimal variation across different annealing temperatures, with noticeable changes only at 300°C, where the surface potential becomes comparable to that of EtOH‐MoS_2_. These temperature‐dependent behaviors indicate that ethanol confinement induces thermally activated electron doping via partial oxidation within vdW gaps, whereas water does not; at 300°C, both systems converge as confined molecules are removed and the structure reverts to the 2H phase. X‐ray photoelectron spectroscopy (XPS) analysis further supports this electron doping effect. The Mo 3d core levels of EtOH‐MoS_2_ exhibit a redshift of approximately −0.4 eV compared to H_2_O‐MoS_2_ (Figure S11), accompanied by a similar shift in the S 2p spectra (Figure S12), confirming an upward shift of the Fermi level toward the conduction band [43, 44].

Figure 2f summarizes the calculated work functions. H_2_O‐MoS_2_ exhibits only minor changes in work function upon annealing, whereas EtOH‐MoS_2_ shows a substantial reduction of ∼0.5 eV after annealing at 200°C, consistent with enhanced electron doping. Thickness‐dependent I–V measurements using the same Pt/Ti IDE geometry show consistently higher resistance for EtOH‐MoS_2_ than for H_2_O‐MoS_2_ at identical nominal thicknesses (Figure S13). This difference is consistent with weaker electrical coupling resulting from the retained intercalant and increased interlayer spacing after annealing at 200°C (1.0 nm for EtOH‐MoS_2_ compared with 0.62 nm for H_2_O‐MoS_2_), as observed by both XRD and HRTEM (Figure 2b and Figure S4).

To evaluate how molecular confinement influences device functionality, we systematically compared the gas sensing characteristics of EtOH‐MoS_2_ and H_2_O‐MoS_2_ under various conditions. Our MoS_2_ sensors were fabricated using a vacuum filtration assembly process, yielding films with high thickness uniformity and minimal device‐to‐device variability. To ensure statistical reliability, at least five independent devices (N = 5) were fabricated and measured under each condition, demonstrating consistent sensing behaviors across all samples. The intrinsic sensing behavior under dry conditions (RH = 0%) was examined to establish a baseline for phase‐dependent reactivity (Figure 3a). Both materials exhibit negligible NO_2_ responses when dominated by the metallic 1T phase, indicating its limited activity for NO_2_ sensing. As the phase evolves toward the semiconducting 2H phase, the response increases and reaches a maximum in the 2H‐dominant regime. Under these conditions, EtOH‐MoS_2_ achieves a maximum response of 14.2%, whereas H_2_O‐MoS_2_ reaches only 5.3%, corresponding to a 268% enhancement enabled by ethanol confinement. These results indicate that while the semiconducting 2H phase governs NO_2_ reactivity, vdW‐gap molecular confinement further amplifies the phase‐dependent response. Specifically, electron doping in EtOH‐MoS_2_ increases the baseline carrier density and facilitates charge transfer upon NO_2_ adsorption, resulting in enhanced response compared to H_2_O‐MoS_2_. This interpretation is further supported by the annealing‐temperature dependence of the NO_2_ response (Figure S14). The response of both films is negligible in the 1T‐dominant regime below 100°C, increases through the mixed‐phase regime, and reaches a maximum at 200°C, where EtOH‐MoS_2_ retains ethanol‐derived species within the vdW gaps, resulting in the greatest enhancement over H_2_O‐MoS_2_. The response of both films then decreases sharply at 300°C, coincident with the complete removal of the confined species and progressive oxidation to MoO_x_.

**FIGURE 3:**
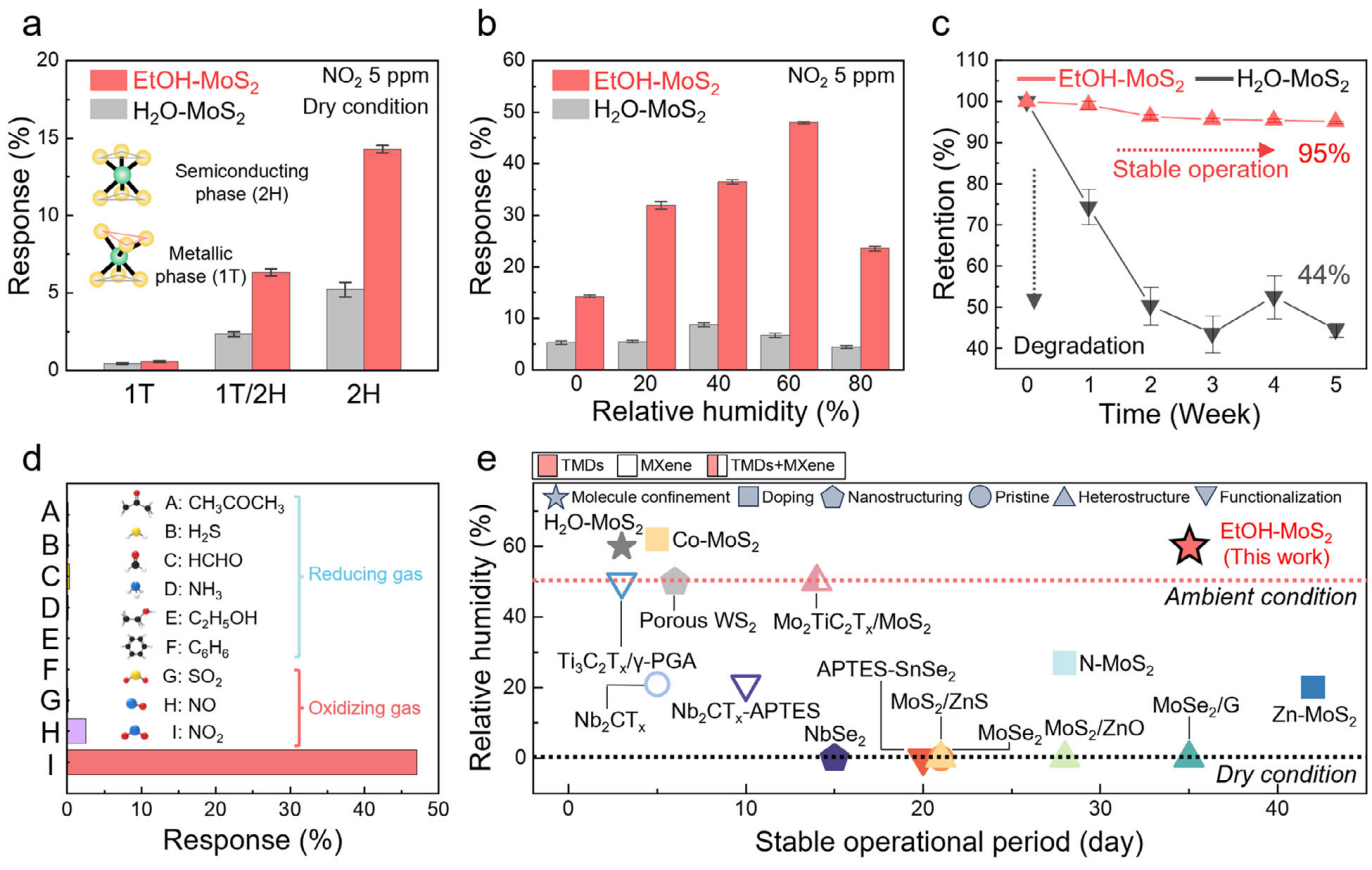
NO_2_ sensing performance of EtOH‐MoS_2_ and H_2_O‐MoS_2_. (a) NO_2_ responses as a function of phase under dry conditions (RH = 0%), highlighting the combined effects of phase and molecular confinement. (b) NO_2_ response as a function of relative humidity (RH 0% –80%), revealing distinct differing humidity tolerance. (c) Long‐term stability of NO_2_ sensing over 5 weeks, demonstrating the superior durability of EtOH‐MoS_2_ compared to H_2_O‐MoS_2_. Retention (%) denotes the sensor response normalized to the initial value. (d) Selectivity of EtOH‐MoS_2_ toward NO_2_ against reducing gases (CH_3_COCH_3_, H_2_S, HCHO, NH_3_, C_2_H_5_OH, C_6_H_6_) and oxidizing gases (NO, SO_2_) measured at 60% RH. (e) Benchmarking of long‐term stability of NO_2_ sensing performance of EtOH–MoS_2_ under dry and ambient conditions against previously reported chemirisistive and electrochemical NO_2_ sensors. Error bars in (a–c) represent the standard deviation of five independent devices measured for each film.

Building on this baseline behavior, we next evaluate the sensing performance under humid conditions (Figure 3b and Figure S15). EtOH‐MoS_2_ exhibits a continuous increase in NO_2_ response with increasing RH, reaching an optimal response of 48% at 60% RH, whereas H_2_O‐MoS_2_ shows only a modest enhancement. At 60% RH, the NO_2_ response of EtOH‐MoS_2_ is approximately seven times higher than that of H_2_O‐MoS_2_. The increase in response with humidity is attributed to adsorbed water molecules that promote charge transfer during NO_2_ adsorption. The superior performance of EtOH‐MoS_2_ relative to H_2_O‐MoS_2_ arises from surface modification induced by ethanol confinement, which suppresses competitive water adsorption and preserves active sites for NO_2_ adsorption under humid conditions. At 80% RH, the response decreases to 23.4%, reflecting a crossover from water‐assisted promotion of NO_2_ adsorption to water‐film‐induced site blocking near saturation, where the surface water coverage transitions from a dispersed sub‐monolayer to a continuous multilayer film that impedes NO_2_ access to the vacancy sites. This value nevertheless remains well above the dry‐condition response of 14.2% and exceeds that of H_2_O‐MoS_2_ at every humidity level. The intrinsic effect of humidity on the surface is further seen in the base resistance measured without target gas (Figure S16). The resistance rises with humidity and then reverses, and this reversal occurs at a higher relative humidity for EtOH‐MoS_2_ (60% RH) than for H_2_O‐MoS_2_ (40% RH), indicating that the ethanol confinement delays the formation of a continuous surface water layer to higher humidity levels.

We then assess the long‐term operational stability under humid conditions (60% RH) to evaluate practical device reliability (Figure 3c and Figure S17). Over the 5 week period, H_2_O‐MoS_2_ exhibits significant degradation, retaining only 44% of its initial response. In contrast, EtOH‐MoS_2_ maintains 95% of its original response, demonstrating that the sensing function is retained under sustained humid exposure rather than merely being less affected by humidity. The high density of sulfur vacancies introduced by chemical exfoliation renders the basal plane chemically reactive, so that the environmental stability associated with pristine MoS_2_ does not apply to either film. The markedly different retention of the two films, despite their comparable vacancy density, indicates that factors beyond vacancy density govern their long‐term operational stability. Additional measurements further confirm the robustness of the EtOH‐MoS_2_ device, including stable baseline resistance without drift (Figure S18) and consistent responses over 50 consecutive NO_2_ (1 ppm) exposure cycles (Figure S19). These results demonstrate reliable long‐term operation under prolonged humid conditions, which is rarely addressed in previous studies, highlighting the practical viability of vdW‐gap‐confined MoS_2_ sensors for real‐world applications.

To evaluate intrinsic sensing capability, concentration‐dependent measurements were performed under optimal humidity conditions. EtOH‐MoS_2_ exhibits a clear linear response over the range of 1–5 ppm (Figure S20a), enabling accurate quantification of NO_2_ concentration, whereas H_2_O‐MoS_2_ shows a significantly weaker response. Additional measurements in the sub‐ppm range (200–800 ppb) using a dedicated gas delivery system show a distinguishable and linear response down to 200 ppb (Figure S20b). From these measurements, the limit of detection (LOD) is estimated to be 36.2 ppb for EtOH‐MoS_2_ and 198 ppb for H_2_O‐MoS_2_ (Figure S21), demonstrating the substantially higher detection sensitivity of EtOH‐MoS_2_.

The selectivity of the sensor was further evaluated against various interfering gases at 60% RH (Figure 3d and Figure S22). EtOH‐MoS_2_ exhibits negligible responses to the reducing gases CH_3_COCH_3_, H_2_S, HCHO, NH_3_, C_2_H_5_OH, and C_6_H_6_, and to the oxidizing gases SO_2_ and NO, demonstrating strong selectivity toward NO_2_ against both classes of interferents. Even NO, which is chemically closest to NO_2_, elicits a response of only 2.5% compared with 48% for NO_2_ under the same conditions. Mixed‐gas measurements (NO_2_ with CH_3_COCH_3_ and HCHO, and NO_2_ with NH_3_ and H_2_S; Figure S23) further confirm minimal interference, highlighting robust NO_2_ detection in complex environments.

Finally, Figure 3e benchmarks the NO_2_ sensing performance of EtOH‐MoS_2_ against previously reported sensors, categorized by material systems and structural engineering strategies (Table S1). The comparison is based on the stable operation time, defined as the duration over which the sensing response remains within ±5% of its initial value, and the relative humidity (RH) under which long‐term stability was evaluated. This benchmark reveals that only a limited number of studies assess long‐term stability under humid conditions, whereas most reports validate performance exclusively under dry or low‐humidity environments. Such evaluations do not adequately represent realistic operating environments, where ambient humidity levels (RH ≥ 50%) [17, 45] are commonly encountered. In practice, sustained operation under humid conditions is often hindered by material degradation, baseline drift, and irreversible water adsorption.

Against this backdrop, EtOH‐MoS_2_ demonstrates markedly improved performance under realistic conditions. While previous approaches have enhanced response through structural engineering (e.g., edge‐rich morphologies, high porosity, or hierarchical architectures) [34, 46], they often suffer from reduced selectivity and stability in the presence of moisture due to non‐specific adsorption.

In contrast, EtOH‐MoS_2_ maintains stable NO_2_ sensing for up to 35 days under high humidity, outperforming previously reported sensors tested under comparable conditions. These results highlight that vdW‐gap molecular confinement provides an effective strategy to simultaneously enhance humidity tolerance and long‐term stability, which are critical for practical NO_2_ sensing.

To elucidate the origin of the highly NO_2_‐selective behavior of EtOH‐MoS_2_ under humid conditions, we performed first‐principles calculations based on experimentally relevant structural models. Our experimental results indicate that the sensing characteristics cannot be explained solely by the sulfur‐vacancy density, suggesting that additional atomic‐scale factors govern the adsorption behavior. We therefore employed first‐principles calculations to identify the microscopic origin of the enhanced sensing performance.

Sulfur vacancies (V_s_) in MoS_2_ are well known as highly reactive sites for gas adsorption [23, 47] and serve as the primary active sites for NO_2_ sensing in both H_2_O‐MoS_2_ and EtOH‐MoS_2_. XPS analysis confirms comparable sulfur vacancy densities in both systems (S/Mo atomic ratios of 1.75 and 1.74 for H_2_O‐MoS_2_ and EtOH‐MoS_2_, respectively, compared with 1.95 for bulk MoS_2_; Figure S24). We therefore investigated whether differences in the atomic‐scale configuration of sulfur vacancies could account for the distinct sensing behaviors of the two systems.

To investigate the atomic‐scale origin of this behavior, we constructed a defective MoS_2_ monolayer with the experimentally relevant vacancy density (∼12.5%) and identified the most energetically stable vacancy configurations via formation energy calculations [48, 49] (Figure S25 and Tables S2 and S3). This monolayer was then used to construct multilayer slab models for both H_2_O‐MoS_2_ and EtOH‐MoS_2_. For EtOH‐MoS_2_, intercalated species were explicitly introduced into the vdW gaps based on experimental evidence. The C═O peak in C 1s XPS spectra (Figure 2d) and the inner‐film carboxylate‐range component observed by depth‐profile XPS (Figure S9) support the presence of an oxidized ethanol‐derived species within the interlayers. Among ethanol, acetaldehyde, and acetate, only acetate has a negative calculated intercalation energy (−1.82 eV, compared with +0.10 eV for ethanol and +0.54 eV for acetaldehyde; Figure S26), justifying its use as the representative model among the three candidates considered. The most stable configuration involves acetate molecules chemically bonded to sulfur vacancies within each MoS_2_ layer (Figure S27), forming a thermally robust interlayer framework [50, 51].

Building on these structural models, we next examined how vacancy configuration governs gas adsorption selectivity. Three representative configurations were considered: isolated vacancies, lined vacancies (two or more adjacent vacancies), and clustered vacancies (three or more aggregated vacancies) because sulfur vacancies tend to aggregate as their local density increases [52, 53] (Figure S28). As shown in Figure 4a, NO_2_ exhibits strong chemisorption (∼−3.0 eV) across all configurations (−2.94, −3.01, and −3.03 eV for isolated, lined, and clustered V_s_, respectively), with the oxygen atom binding at the center of a triangular Mo arrangement in each case [54]. In contrast, H_2_O adsorption strongly depends on vacancy configuration. H_2_O binds weakly to isolated and lined V_s_ (−0.15 and −0.18 eV, respectively), but much more strongly to clustered V_s_ (−1.15 eV).

**FIGURE 4:**
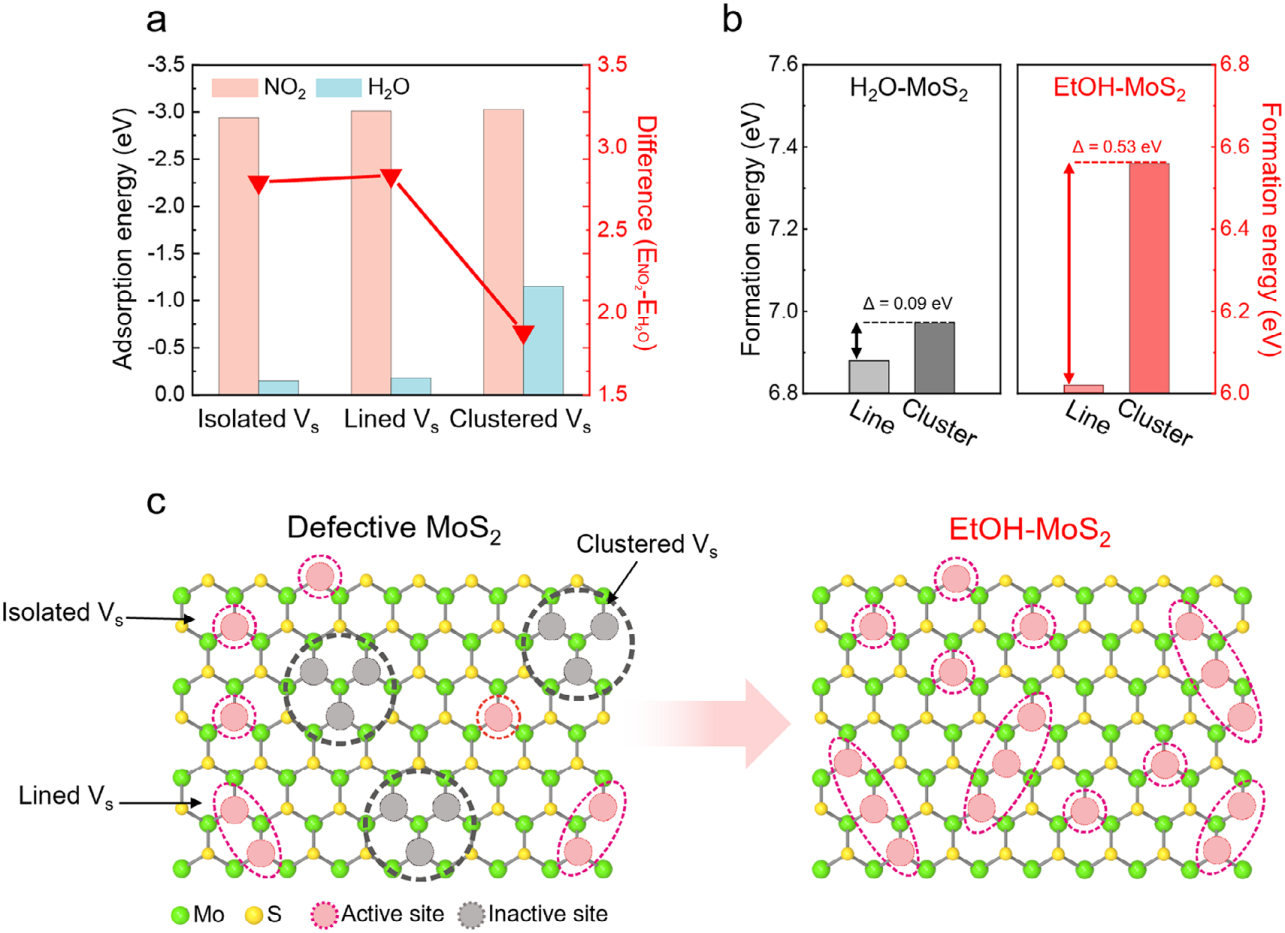
Vacancy configuration‐dependent mechanism for enhanced NO_2_ response and humidity stability of H_2_O‐MoS_2_ and EtOH‐MoS_2_ films. (a) Calculated adsorption energies of NO_2_ and H_2_O depending on different sulfur vacancy (V_s_) configurations. (b) Formation energy differences between lined and clustered V_s_ in H_2_O‐MoS_2_ and EtOH‐MoS_2_. (c) Schematic illustration of vacancy configurations and their impact on gas adsorption: isolated and lined V_s_ serve as active sites for NO_2_ adsorption, whereas clustered V_s_ are less effective. Molecular confinement suppresses vacancy clustering and promotes the formation of reactive configurations.

This difference originates from the local coordination environment of Mo atoms. In isolated and lined V_s_, Mo sites are partially screened by surrounding electronegative S atoms, whose lone pairs repel the oxygen end of H_2_O and suppress adsorption. The marginal energy difference between these two configurations reflects their similarly sufficient S coordination. However, clustered V_s_ exhibit a higher degree of Mo under‐coordination, which reduces this screening effect and enhances the local electrostatic potential, leading to stronger attraction toward polar H_2_O molecules (Figures S29 and S30 and Table S4). A consistent trend is observed for EtOH‐MoS_2_ (Figures S31 and S32), confirming the generality of this behavior. As a result, the energy difference favoring NO_2_ over H_2_O decreases from 2.79 and 2.83 eV for the isolated and lined Vs to 1.88 eV for the clustered V_s_, indicating a substantially reduced NO_2_/H_2_O selectivity for the clustered configuration due to its enhanced water affinity.

To evaluate how molecular confinement modifies vacancy formation energetics, we calculated the formation energies of lined and clustered V_s_ in both H_2_O‐MoS_2_ and EtOH‐MoS_2_ (Figure 4b). In H_2_O‐MoS_2_, lined V_s_ are only marginally more stable than clustered V_s_ (ΔE = 0.09 eV), consistent with prior studies [55, 56]. This small energy difference indicates the coexistence of both configurations, including clustered V_s_ that degrade selectivity. In contrast, EtOH‐MoS_2_ exhibits a significantly larger energy difference (ΔE = 0.53 eV), indicating that molecular confinement strongly suppresses the formation of clustered V_s_. This stabilization arises from the steric and electronic effects of the confined partially oxidized ethanol‐derived species (represented by acetate in the calculations), which locally constrain the interlayer structure and increase the energetic penalty of vacancy aggregation. Consequently, EtOH‐MoS_2_ preferentially forms isolated and lined V_s_, which combine strong NO_2_ affinity with weak H_2_O interaction. This provides a direct mechanistic explanation for its enhanced NO_2_ selectivity and humidity tolerance compared to H_2_O‐MoS_2_. These computational results are fully consistent with the experimental observations, including the confinement‐induced vdW gap expansion revealed by XRD and HRTEM, the comparable sulfur‐vacancy density determined by XPS, and the distinct NO_2_ selectivity and humidity stability of the two films.

As illustrated in Figure 4c, ethanol‐derived intercalants redistribute sulfur vacancies by suppressing clustered configurations and favoring isolated and lined arrangements, thereby preserving selective active sites even under high‐humidity conditions. Collectively, these results establish a clear mechanistic basis for the enhanced NO_2_ sensing performance of EtOH‐MoS_2_, highlighting its potential for practical gas sensing in moisture‐rich environments.

## 3 Conclusion

In summary, we demonstrate a vdW‐gap‐confined molecular engineering strategy for MoS_2_ that enables simultaneous enhancement of response and selectivity, together with humidity‐tolerant gas sensing. This approach, based on solution‐processable assembly, does not rely on extrinsic dopants or post‐synthetic treatments, offering a scalable and versatile platform for layered 2D materials.

By confining small molecules within vdW gaps, the surface charge environment of MoS_2_ is modulated, resulting in reduced work function and electron doping that enhances charge transfer and improves response toward electron‐withdrawing gases such as NO_2_. Beyond electronic modulation, molecular confinement also governs the spatial configuration of V_s_, which serves as a critical active site for gas adsorption. In particular, this strategy suppresses clustered V_s_ that promote non‐selective water adsorption, while favoring isolated and lined V_s_ that enable selective NO_2_ adsorption. This vacancy configuration control provides a direct mechanistic origin for the enhanced selectivity and humidity tolerance observed experimentally. In addition, molecular confinement modulates surface wettability, reducing water affinity and further contributing to humidity‐resilient sensing.

More broadly, our results establish vdW‐gap molecular confinement as a strategy for dual control of electronic structure and defect configuration, which govern response and selectivity, respectively. This synergistic modulation defines a new design paradigm for gas sensors, enabling high‐performance operation under humid conditions and offering a pathway toward practical, moisture‐resilient sensing technologies based on 2D materials.

## 4 Experimental Section

### 4.1 Synthesis of MoS_2_ Nanosheets and Phase‐Engineering

Bulk 2H‐MoS_2_ was converted to the metallic 1T phase via lithium intercalation [13, 38] by reacting 20 mg of MoS_2_ powder (Sigma–Aldrich) with 2 mL of n‐butyllithium in hexane (1.6 M, Sigma–Aldrich) in a Teflon‐lined autoclave under an argon atmosphere. The resulting Li_x_MoS_2_ reacted with water to generate LiOH and hydrogen gas, inducing exfoliation into monolayer nanosheets dispersed in water. The dispersion was centrifuged to remove unexfoliated aggregates, and the supernatant containing monolayer nanosheets was collected as the stock dispersion at a concentration of 1 mg mL^−1^. Due to the metastable nature of the 1T phase, it gradually reverted to the thermodynamically stable 2H phase upon thermal annealing between room temperature and 300°C. The phase evolution and structural characteristics were examined using spectroscopic and microscopic techniques.

### 4.2 Synthesis of Molecule‐Confined MoS_2_ Films

To prepare freestanding MoS_2_ films, a precursor solution was formulated by mixing the aqueous dispersion of exfoliated MoS_2_ nanosheets with a target molecule‐rich solvent. This mixture ensures that the target molecules are present in excess, enabling their confinement between nanosheets during film formation. The stock dispersion was diluted 25‐fold with the target solvent to a working concentration of 0.04 mg mL^−1^. The resulting solution was filtered through an anodized aluminum oxide membrane (Anodisc, 0.2 µm pore size) using a vacuum filtration apparatus. During the process, the solvent molecules pass through the membrane while the MoS_2_ nanosheets are retained on the surface, where they re‐stack into a plane‐to‐plane network, forming a uniform film whose thickness is determined by the filtered volume at a fixed dispersion concentration. The film was then transferred onto the interdigitated electrodes. The thickness uniformity and film‐to‐film reproducibility were assessed by AFM.

The type of molecule confined within the van der Waals (vdW) gaps is determined by the solvent used during filtration. Depending on the solvent species, different molecules can be incorporated between MoS_2_ layers, thereby influencing the interlayer spacing and surface properties of the resulting film.

### 4.3 Materials Characterization

Raman spectroscopy was conducted using a custom‐built setup equipped with a 532 nm excitation laser and a monochromator (Andor, SOLIS 303i). The 2H phase of MoS_2_ is typically identified by its two prominent Raman modes: the in‐plane E^2^_1g_ mode at 383 cm^−^^1^ and the out‐of‐plane A_1g_ mode at 409  cm^−1^. In contrast, the 1T metallic phase exhibits distinct J‐series modes, specifically J_1_ (156 cm^−1^), J_2_ (228  cm^−1^), and J_3_ (330  cm^−1^). In this study, phase transitions were monitored by tracking the appearance or disappearance of these characteristic vibrational modes associated with each crystalline phase.

The thickness and surface morphology of the films were analyzed using atomic force microscopy (AFM, Park Systems, XE‐10) in non‐contact mode.

To investigate the electronic structure and surface potential distribution, Kelvin probe force microscopy (KPFM) measurements were performed using the same AFM system equipped with a conductive tip (NSC36/Cr‐Au, Park Systems). Prior to measurements, the system was calibrated using highly ordered pyrolytic graphite (HOPG) as a reference due to its well‐defined and stable work function. The surface potential, defined as the contact potential difference (CPD) between the tip and the sample, was calculated using the following relation:

$$V_{CPD}=\frac{\phi_{tip}-\phi_{sample}}{e}$$

where ϕ_*tip*_ and ϕ_*sample*_ are the work functions of the tip and the sample, respectively, and *e* is the elementary charge. An increase in the measured surface potential (i.e., more positive *V_CPD_*) corresponds to a decrease in the sample's work function, implying an upward shift of the Fermi level. This behavior is indicative of electron doping, where the enhanced electron concentration shifts the Fermi level closer to the conduction band.

To analyze the chemical composition and monitor electronic changes associated with the phase transition, x‐ray photoelectron spectroscopy (XPS) was performed using an X‐tool system (ULVAC‐PHI) equipped with a monochromatic Al Kα x‐ray source (1486.6 eV) under ultrahigh vacuum (∼10^−10^ Torr). Binding energies were calibrated to the C 1s peak at 284.4 eV. The Mo 3d and S 2p phase and doping assignments were determined from the measured peak positions, whereas the O 1s spectra were fitted with Voigt profiles following Shirley background subtraction, using five components (Mo─O, C═O, adsorbed H_2_O, C─O, and gaseous H_2_O) with peak positions constrained within 0.2 eV and FWHM within 1.6 to 2.0 eV. The S/Mo atomic ratios were obtained from integrated peak areas normalized by relative sensitivity factors. Depth‐profile XPS was additionally performed on both films after annealing at 200°C. The sputter‐time axes were converted to thickness‐anchored nominal depth scales, with each film/substrate boundary identified from the increase in normalized Si 2p intensity. The surface interval was defined as 0–1.5 nm, and the inner‐film windows were defined as 1.5–24 nm for EtOH‐MoS_2_ and 1.5–21 nm for H_2_O‐MoS_2_. The C/Mo XPS peak‐area ratio was calculated from the integrated C 1s and Mo 3d peak areas. The C 1s spectra were referenced to the C–C component at 284.4 eV, fitted with Voigt profiles after Shirley background subtraction, and averaged within the defined depth windows.

To examine changes in the interlayer spacing and crystallographic structure, x‐ray diffraction (XRD) analysis was conducted using a SmartLab diffractometer (Rigaku) with Cu Kα radiation (λ = 0.15412 nm). The interlayer spacing was calculated using Bragg's law (*n*λ  = 2d sin(θ) ), where d is the spacing between adjacent layers, *λ* is the x‐ray wavelength, and θ is the diffraction angle. Upon lithium intercalation, the (002) peak characteristic of the 2H phase (∼14.4°) shifted to a higher angle with broadening, indicative of structural distortion. In addition, a new diffraction peak appeared below 10°, corresponding to an expanded van der Waals gap caused by intercalated lithium ions or molecules. The interlayer distance was estimated based on the position of this low‐angle peak.

To examine the interlayer spacing, cross‐sectional high‐resolution transmission electron microscopy (HRTEM) images were acquired on a Tecnai F20 operated at 200 kV.

### 4.4 Fabrication of Sensor Device

Pt/Ti interdigitated electrodes (IDEs) with thicknesses of 80 and 20 nm, respectively, were fabricated on a 4‐inch SiO_2_/Si substrate (1 µm/550 µm thick). The Pt/Ti layers were deposited using an e‐beam evaporator, and the electrode patterns were defined through photolithography, followed by reactive ion etching. Each electrode was spaced 50 µm apart, with a total of 12 electrodes arranged within a 1.5 mm × 1.5 mm area. The patterned Pt‐IDEs were sequentially cleaned with acetone and isopropyl alcohol, then dried under a nitrogen gas flow.

### 4.5 Gas Sensing Measurements

The gas sensing performance of EtOH‐MoS_2_ and H_2_O‐MoS_2_ was assessed in a quartz tube at room temperature. A mass‐flow controller (MFC) system, incorporating a bubbler, was used to regulate the gas flow by mixing dry air with water vapor, allowing the relative humidity (RH) to be adjusted from 0% to 80% while maintaining a constant flow rate of 1000 sccm. The sensor resistance was monitored under a 0.5 V DC bias voltage using a Keithley 2401 instrument, and all data were collected on a computer via the general‐purpose interface bus (GPIB) using LabVIEW software. The sensor response was calculated using the equation:

$$\mathrm{Response}\left(\%\right)=\left(\frac{R_{a}}{R_{g}}-1\right)\times 100$$

### 4.6 Method of Computational Calculation

All first principles calculations were performed with the Vienna ab initio simulation package (VASP) based on density functional theory (DFT). For the core electrons to pseudopotentials, the projector‐augmented wave (PAW) method is implemented. Perdew‐Burke‐Ernzerhof (PBE) functional with generalized gradient approximation (GGA) was applied to describe the exchange‐correlation energy [57, 58, 59]. To properly present the van der Waals interaction of gas molecules, the DFT‐D3 method by Grimme and co‐workers was applied [60, 61]. For the electronic relaxation of the system, an energy cutoff of 400 eV and an energy step convergence cutoff of 10^−5^ eV were used. The geometry of atoms was relaxed to meet the Hellmann‐Feynman forces below 0.01 eV/Å. To mitigate the periodic interaction of vacuum slab cell, a dipole correction along the *c*‐axis, the direction of the vacuum layer, is employed.

## Author Contributions

**Jae Hyung Shim**: Writing – original draft, conceptualization, methodology, visualization, validation, investigation, data curation, formal analysis. **Gwang Su Kim**: conceptualization, writing – original draft, validation, formal analysis, data curation, methodology. **Tae Hyung Lee**: conceptualization, writing – original draft, investigation, visualization, software, formal analysis, data curation. **Jaeho Lee**: validation, formal analysis, investigation. **Su‐Yeon Joung**: validation, methodology, data curation. **Jung Hoon Kang**: investigation, methodology, formal analysis. **Jin Seok Kim**: investigation, validation, formal analysis. **Jung‐Won An**: investigation. **Chong‐Yun Kang**: resources, writing – review and editing. **Ho Won Jang**: resources, writing – review and editing. **Jungwon Park**: resources, writing – review and editing. **Ji‐Soo Jang**: writing – review and editing, conceptualization, project administration, resources, funding acquisition, supervision. **Chul‐Ho Lee**: resources, project administration, writing – review and editing, conceptualization, supervision, funding acquisition.

## Conflicts of Interest

The authors declare no conflicts of interest.

## Supporting information




**Supporting File**: advs78072‐sup‐0001‐SuppMat.docx.

## Data Availability

The data that support the findings of this study are available from the corresponding author upon reasonable request.
