# Supplementary material for: Van der Waals Gap‐Confined Molecular Engineering of MoS2 for Selectivity‐Enhanced and Humidity‐Tolerant Gas Sensing
Source: Adv Sci (Weinh). 2026 Oct 8:e78072. Online ahead of print. doi: 10.1002/advs.78072 (PMC13650019; doi:10.1002/advs.78072)
Supplement: Supplementary file 1 [file ADVS-9999-e78072-s001.docx]

**Supporting Information**

Van der Waals Gap-Confined Molecular Engineering of MoS_2_ for Selectivity-Enhanced and Humidity-Tolerant Gas Sensing

*Jae Hyung Shim^1,2,3†^, Gwang Su Kim^4†^, Tae Hyung Lee^5,6†^, Jaeho Lee^1,2^, Su-Yeon Joung^1,2^, Jung Hoon Kang^1,2^, Jin Seok Kim^1,2^, Jung-Won An^4,5^, Chong-Yun Kang^4^, Ho Won Jang^5,7^, Jungwon Park^6,7,8,9^, Ji-Soo Jang^4,10*^, and Chul-Ho Lee^1,2,3*^*

^1^Department of Electrical and Computer Engineering, Seoul National University, Seoul 08826, Republic of Korea

^2^Inter-University Semiconductor Research Center (ISRC), Seoul National University, Seoul 08826, Republic of Korea

^3^Seoul National University Energy Initiative(SNUEI), Seoul National University, Seoul 08826, Republic of Korea

^4^Electronic and Hybrid Materials Research Center, Korea Institute of Science and Technology (KIST), Seoul 02792, Republic of Korea

^5^Department of Materials Science and Engineering, Seoul National University, Seoul 08826, Republic of Korea

^6^Department of Chemical and Biological Engineering, Seoul National University, Seoul 08826, Republic of Korea

^7^Advanced Institutes of Convergence Technology, Seoul National University, Gyeonggi-do 16229, Republic of Korea

^8^Center for Nanoparticle Research, Institute for Basic Science (IBS), Seoul 08826, Republic of Korea

^9^Institute of Engineering Research, College of Engineering, Seoul National University, Seoul 08826, Republic of Korea

^10^SKKU Advanced Institute of Nanotechnology and Department of Nanoengineering, Sungkyunkwan University, Suwon 16419, Republic of Korea

**Corresponding author e-mail:* *[chulholee@snu.ac.kr](mailto:chulholee@snu.ac.kr), wkdwltn92@skku.edu*

**Contents**

Figure S1. Fabrication process of the Ethanol-confined MoS_2_ and Water-confined MoS_2_ films.

Figure S2. Thickness uniformity and reproducibility of EtOH-MoS_2_ films assessed by AFM.

Figure S3. Water contact angle of EtOH-MoS_2_ and H_2_O-MoS_2_ films in the as-prepared state and after annealing.

Figure S4. Interlayer spacing of molecule-confined MoS_2_ films.

Figure S5. XPS Mo 3d spectra of (a) EtOH-MoS_2_ and (b) H_2_O-MoS_2_ films in the as-prepared state and after annealing.

Figure S6. Raman spectra of (a) EtOH-MoS_2_ and (b) H_2_O-MoS_2_ films in the as-prepared state and after annealing.

Figure S7. Evolution of the Mo 3d components with annealing temperature.

Figure S8. XPS C 1s spectra of (a) EtOH-MoS_2_ and (b) H_2_O-MoS_2_ films in the as-prepared state and after annealing.

Figure S9. XPS depth profiles of EtOH-MoS_2_ and H_2_O-MoS_2_ films annealed at 200 °C.

Figure S10. Temperature-dependent KPFM surface potential mapping and histogram analysis of EtOH-MoS_2_ and H_2_O-MoS_2_ films.

Figure S11. Comparison of EtOH-MoS_2_ and H_2_O-MoS_2_ in Molybdenum (Mo) peaks via XPS.

Figure S12. Comparison of EtOH-MoS_2_ and H_2_O-MoS_2_ in sulfur (S) peaks via XPS.

Figure S13. Thickness-dependent I–V characteristics of EtOH-MoS_2_ and H_2_O-MoS_2_ films measured using the same Pt/Ti interdigitated-electrode geometry.

Figure S14. NO_2_ response of H_2_O-MoS_2_ and EtOH-MoS_2_ after annealing at various temperatures (25–300 °C) under dry conditions (RH 0%).

Figure S15. Dynamic NO_2_ sensing behavior of (a) EtOH-MoS_2_ and (b) H_2_O-MoS_2_ films under varying relative humidity from 0 to 80% RH.

Figure S16. Relative changes in base resistance of EtOH-MoS_2_ and H_2_O-MoS_2_ sensors as a function of relative humidity (RH).

Figure S17. Time-dependent evolution of NO_2_ sensing response for (a) EtOH-MoS_2_ and (b) H_2_O-MoS_2_ from 0 to 5 weeks.

Figure S18. Long-term stability assessment of EtOH-MoS_2_ and H_2_O-MoS_2_ from 0 to 5 weeks.

Figure S19. Repeatability and durability assessment for EtOH-MoS_2_.

Figure S20. Dynamic NO_2_ sensing response of EtOH-MoS_2_ at RH 60%.

Figure S21. Sensitivity plot of EtOH-MoS_2_ and H_2_O-MoS_2_ for NO_2_ concentration variation.

Figure S22. Selectivity of EtOH-MoS_2_ toward NO_2_ against oxidizing and reducing interferents.

Figure S23. Mixed gas measurements for EtOH-MoS_2_.

Figure S24. Comparison of atomic ratio of molecule-confined MoS_2_ films.

Figure S25. Vacancy Formation in Monolayer MoS_2_.

Figure S26. Intercalation energy per intercalant molecule.

Figure S27. Slab cell of MoS_2_ constructed with monolayer MoS_2_ with 4 S vacancies.

Figure S28. Surface S vacancy configuration in pristine MoS_2_ and acetic acid intercalated MoS_2_.

Figure S29. H_2_O and NO_2_ binding structure on pristine MoS_2_ with surface line S vacancies.

Figure S30. H_2_O and NO_2_ binding structure on pristine MoS_2_ with surface clustered S vacancies.

Figure S31. H_2_O and NO_2_ binding structure on EtOH intercalated MoS_2_ with surface lined S vacancies.

Figure S32. H_2_O and NO_2_ binding structure on EtOH intercalated MoS_2_ with surface clustered S vacancies.

Table S1. Comparison of reported 2D chemiresistive NO_2_ sensing materials at RT.

Table S2. Lattice parameter of relaxed bulk MoS_2_

Table S3. Vacancy Formation Energy in Monolayer MoS_2_

Table S4. NO_2_ and H_2_O adsorption energy at Lined and Clustered Vs on H_2_O-MoS_2_ and EtOH-MoS_2_.

**Figure S1. Fabrication process of the Ethanol-confined MoS_2_ and Water-confined MoS_2_ films.** During this process, monolayer-dispersed MoS_2_ flakes, stabilized by their strong negative surface charge, self-assemble into a continuous film. Solvent molecules become entrapped between the MoS_2_ layers during filtration and remain confined upon natural drying, forming large-area films with interlayer molecular confinement. Films synthesized with intercalated water or ethanol are referred to as (a) EtOH-MoS_2_ and (b) H_2_O-MoS_2_, respectively.

**Figure S2. Thickness uniformity and reproducibility of EtOH-MoS_2_ films assessed by AFM.** (a-d) AFM topography images and the corresponding height profiles measured along four line scans at different positions on each of four films (#1-#4) prepared separately under identical conditions, showing uniform thickness within each film. (e) Statistical distribution of the thickness values compiled from the line profiles, with mean values of 20.2, 20.3, 24.7, and 22.9 nm for films #1 to #4. The distributions confirm that films of consistent thickness in the 20 to 25 nm range are reproducibly obtained by concentration-fixed vacuum filtration.

**Figure S3. Water contact angle of EtOH-MoS_2_ and H_2_O-MoS_2_ films in the as-prepared state and after annealing at 100, 200, and 300 °C.** EtOH-MoS_2_ exhibits a non-monotonic evolution with a maximum of 66.1 degrees at 200 °C followed by a decrease to 59.8 degrees at 300 °C, whereas H_2_O-MoS_2_ increases monotonically to 58.2 degrees, so that the two films converge at 300 °C. Error bars represent the standard deviation of five measurements at different positions on each film.

**Figure S4. Interlayer spacing of molecule-confined MoS_2_ films.** (a) XRD patterns of EtOH-MoS_2_ and (b) H_2_O-MoS_2_ films in the as-prepared state and after annealing at 100 to 300 °C, with schematic illustrations of the corresponding interlayer structures. The positions of the (001) and (002) peaks shift with annealing temperature, reflecting the evolution of the interlayer spacing upon removal of the confined molecules. (c) Cross-sectional HRTEM images of EtOH-MoS_2_ (left) and H_2_O-MoS_2_ (right) annealed at 200 °C, with the corresponding intensity line profiles yielding interlayer spacings of 1.0 and 0.62 nm, in close agreement with the XRD values and directly visualizing the retention of the expanded van der Waals gap in the ethanol-confined film.

**Figure S5. XPS Mo 3d spectra of (a) EtOH-MoS_2_ and (b) H_2_O-MoS_2_ films in the as-prepared state and after annealing at 50 to 300 °C.** The Mo 3d peaks shift from the 1T positions to the 2H positions with increasing annealing temperature, indicating the 1T-to-2H phase evolution. Both films become predominantly 2H after annealing at 200 °C and above.

**Figure S6. Raman spectra of (a) EtOH-MoS_2_ and (b) H_2_O-MoS_2_ films in the as-prepared state and after annealing at 50 to 300 °C.** The J_1_ mode characteristic of the 1T phase is present in the as-prepared films and disappears upon annealing, while the 2H-phase modes $E_{2g}^{1}$ (in-plane) and $A_{1g}$ (out-of-plane) become progressively more pronounced. Both films therefore exhibit a temperature-driven evolution from a 1T-rich to a 2H-rich state.

**Figure S7. Evolution of the Mo 3d components with annealing temperature.** Spectral-area fractions of 1T-MoS_2_, 2H-MoS_2_ and MoO_x_ for (a) EtOH-MoS_2_ and (b) H_2_O-MoS_2_ films. The 25 °C points correspond to the as-prepared films.

**Figure S8. XPS C 1s spectra of (a) EtOH-MoS_2_ and (b) H_2_O-MoS_2_ films in the as-prepared state and after annealing at 50 to 300 °C.** EtOH-MoS_2_ exhibits a pronounced C=O component near 288.3 eV that is nearly absent in H_2_O-MoS_2_, consistent with partially oxidized ethanol-derived species remaining confined within the van der Waals gaps. The evolution of the C 1s components with annealing temperature reflects the presence and chemical state of the confined species.

**Figure S9. XPS depth profiles of EtOH-MoS_2_ and H_2_O-MoS_2_ films annealed at 200 °C.** (a) Film/SiO_2_ geometry. (b) C 1s/Mo 3d peak-area ratio and normalized Si 2p intensity versus nominal sputter depth. Vertical dashed lines delimit the surface, inner-film, and substrate regions, and horizontal dashed lines indicate inner-film means of 0.108 ± 0.029 for EtOH-MoS_2_ and 0.050 ± 0.017 for H_2_O-MoS_2_ (mean ± SD across the inner-film depth points). (c, d) Surface C 1s spectrum at 1.5 nm and inner-film spectrum averaged over 1.5–24 nm for EtOH-MoS_2_. (e, f) Corresponding spectra at 1.5 nm and averaged over 1.5–21 nm for H_2_O-MoS_2_.

**Figure S10. Temperature-dependent KPFM surface potential mapping and histogram analysis of EtOH-MoS_2_ and H_2_O-MoS_2_ films.** KPFM measures the contact potential difference (CPD), providing insight into surface-level electronic properties. The CPD maps show a pronounced temperature-dependent shift for EtOH-MoS_2_, whereas H_2_O-MoS_2_ exhibits only minor changes and remains predominantly in the negative-potential (blue) region. In the CPD maps, red and blue correspond to relatively positive and negative surface potential values, respectively. Consistent with the maps, the corresponding histograms summarize the distribution of surface potential values within the selected region. Notably, EtOH-MoS_2_ shows a positive shift in the surface potential distribution at 100 °C and 200 °C, which is not observed for H_2_O-MoS_2_, indicating ethanol-confinement-induced electronic doping at the film surface. The representative values reported in **Figure** **2**e were obtained as the mean surface potential extracted from the CPD maps and the corresponding histograms.

**Figure S11. Comparison of EtOH-MoS_2_ and H_2_O-MoS_2_ in Molybdenum (Mo) peaks via XPS.** The result shows n-type doping behavior through the red shift of Mo 3d peaks.

**Figure S12. Comparison of EtOH-MoS_2_ and H_2_O-MoS_2_ in sulfur (S) peaks via XPS.** The result shows n-type doping behavior through the red shift of S 2p peaks.

**Figure S13. Thickness-dependent I–V characteristics of EtOH-MoS_2_ and H_2_O-MoS_2_ films measured using the same Pt/Ti interdigitated-electrode geometry.** I–V characteristics of EtOH-MoS_2_ (a) and H_2_O-MoS_2_ (b) at nominal thicknesses of 10, 20, and 50 nm. The displayed −1 to +1 V interval includes the 0.5 V sensing bias. The curves are approximately linear and symmetric without pronounced rectification over the displayed range. (c) Resistances measured across the same IDE geometry and obtained from the corresponding I–V curves. The values at 10, 20, and 50 nm are 663, 77.3, and 20.8 MΩ for EtOH-MoS_2_ and 161, 13.1, and 1.9 MΩ for H_2_O-MoS_2_, respectively. These values represent the total resistance measured across the IDEs and include contributions from the film-electrode interfaces and the film network.

**Figure S14. NO_2_ response of H_2_O-MoS_2_ and EtOH-MoS_2_ after annealing at various temperatures (25–300 °C) under dry conditions (RH 0%).** Measurements were performed in the presence of 5 ppm NO_2_ demonstrating the impact of thermal treatment on sensing performance.

**Figure S15. Dynamic NO_2_ sensing behavior of (a) EtOH-MoS_2_ and (b) H_2_O-MoS_2_ films under varying relative humidity from 0 to 80% RH.** Measurements were performed at room temperature in the presence of 5 ppm NO_2_. The resistance decreases upon NO_2_ exposure in both films, and EtOH-MoS_2_ exhibits a markedly larger response with a pronounced humidity dependence, whereas H_2_O-MoS_2_ shows only small changes across the entire humidity range.

**Figure S16. Relative change in base resistance of EtOH-MoS_2_ and H_2_O-MoS_2_ sensors as a function of relative humidity (0 to 80% RH) without target gas exposure.** Each value is the fully equilibrated base resistance measured after sufficient aging at each RH level, normalized to the dry condition (0% RH). Both films show a non-monotonic dependence in which the resistance increases with humidity, reaches a maximum, and then decreases. The maximum occurs at 40% RH for H_2_O-MoS_2_ and at 60% RH for EtOH-MoS_2_, and at 80% RH the resistance change of H_2_O-MoS_2_ falls below its dry-condition value while that of EtOH-MoS_2_ remains positive.

**Figure S17. Time-dependent evolution of NO_2_ sensing response for (a) EtOH-MoS_2_ and (b) H_2_O-MoS_2_ from 0 to 5 weeks.** Measurements were performed at weekly intervals to evaluate long-term sensing stability.

**Figure S18. Long-term stability assessment of EtOH-MoS_2_ and H_2_O-MoS_2_ from 0 to 5 weeks.** The figure presents the temporal evolution of base resistance and sensitivity, highlighting differences in durability and performance retention between (a) EtOH-MoS_2_ and (b) H_2_O-MoS_2_ films.

**Figure S19. Repeatability and durability assessment for EtOH-MoS_2_.** The sensor underwent over 50 consecutive NO_2_ (1 ppm) exposure cycles to evaluate its stability and performance consistency.

**Figure S20. Dynamic NO_2_ sensing response of EtOH-MoS_2_ at 60% RH with concentrations of (a) 1-5 ppm and (b) 200 – 800 ppb.** The response increases with concentration and exhibits a linear dependence within these ranges.

**Figure S21. Response of EtOH-MoS_2_ and H_2_O-MoS_2_ as a function of NO_2_ concentration (1–5 ppm).** EtOH-MoS_2_ exhibits a linear increase in response with rising concentration, while H_2_O-MoS_2_ shows a significantly lower response. The sensitivity, defined as the slope of the linear fit, and the resulting limit of detection (LOD) are indicated.

**Figure S22. Selectivity of EtOH-MoS_2_ toward NO_2_ against oxidizing and reducing interferents.** The responses to the oxidizing gases (NO, SO_2_) and to the reducing gases (CH_3_COCH_3_, H_2_S, HCHO, NH_3_, C_2_H_5_OH, C_6_H_6_) were measured at 5 ppm and 60% RH and compared with the response to NO_2_.

**Figure S23. Mixed gas measurements for EtOH-MoS_2_.** Black: NO_2_ (5 ppm), Red: NO_2_ in the presence of CH_3_COCH_3_ and HCHO (each 5 ppm), Blue: NO_2_ in the presence of NH_3_ and H_2_S (each 5 ppm).

**Figure S24. Comparison of atomic ratio of molecule-confined MoS_2_ films.** Atomic ratio of S/Mo represents the high sulfur vacancy concentration compared to bulk MoS_2_. H_2_O-MoS_2_ and EtOH-MoS_2_ show similar density of sulfur vacancies.

**Figure S25. Vacancy Formation in Monolayer MoS_2_.** (a) Side view of monolayer MoS_2_ vacuum slab. Top view of S vacancy sites with total vacancy number of (b) V_s_2, (c) V_s_3, and (d) V_s_4. (e) Final structure of exfoliated MoS_2_ monolayer with 4 S vacancies. Black shaded circle denotes S vacancy at top S layer and black hollow circle denotes S vacancy at bottom S layer. Red colored circles denote the initial S vacancy configuration.

**Figure S26. Intercalation energy per intercalant molecule of acetate, aldehyde and ethanol in MoS_2_ structure with 12.5% S vacancy.** Intercalation energy is calculated with MoS_2_ structure without intercalant and free intercalant molecules in vacuum slab as reference.

**Figure S27. Slab cell of MoS_2_ constructed with monolayer MoS_2_ with 4 S vacancies.** (a) Pristine MoS_2_ and (b) EtOH intercalated MoS_2_ slab cell structure.

**Figure S28. Surface S vacancy configuration in pristine MoS_2_ and acetate-intercalated MoS_2_.** (a) Lined and (b) clustered surface S vacancies in pristine MoS_2_. (c) Lined and (d) clustered surface S vacancy in EtOH intercalated MoS_2_. Bottom layer S atoms and intercalants are presented as lines.

**Figure S29. H_2_O and NO_2_ binding structure on pristine MoS_2_ with surface line S vacancies.** (a) Side and (b) top view of the NO_2_ binding structure on pristine MoS_2_ with surface line S vacancies. (c) Side and (d) top view of the H_2_O binding structure on pristine MoS_2_ with surface line S vacancies.

**Figure S30. H_2_O and NO_2_ binding structure on pristine MoS_2_ with surface clustered S vacancies.** (a) Side and (b) top view of the NO_2_ binding structure on pristine MoS_2_ with surface clustered S vacancies. (c) Side and (d) top view of the H_2_O binding structure on pristine MoS_2_ with surface clustered S vacancies.

**Figure S31. H_2_O and NO_2_ binding structure on EtOH intercalated MoS_2_ with surface lined S vacancies.** (a) Side and (b) top view of the NO_2_ binding structure on EtOH intercalated MoS_2_ with surface lined S vacancies. (c) Side and (d) top view of the H_2_O binding structure on EtOH intercalated MoS_2_ with surface lined S vacancies.

**Figure S32. H_2_O and NO_2_ binding structure on EtOH intercalated MoS_2_ with surface clustered S vacancies.** (a) Side and (b) top view of the NO_2_ binding structure on EtOH intercalated MoS_2_ with surface clustered S vacancies. (c) Side and (d) top view of the H_2_O binding structure on EtOH intercalated MoS_2_ with surface clustered S vacancies.

**Table S1.** Comparison of reported 2D chemiresistive NO_2_ sensing materials at room temperature

**Table S2**. Lattice parameter of relaxed bulk MoS_2_

**Table S3.** Vacancy Formation Energy in Monolayer MoS_2_

**Table S4.** NO_2_ and H_2_O adsorption energy at Lined and Clustered V_s_ on H_2_O-MoS_2_ and EtOH-MoS_2_.

**Supplementary References**

1. Y. Kim, K. C. Kwon, S. Kang, et al., “Two-Dimensional NbS_2_ Gas Sensors for Selective and Reversible NO_2_ Detection at Room Temperature,” *ACS Sensors* *4*, no. 9 (2019): 2395–2402, https://doi.org/10.1021/acssensors.9b00992.

2. X. Guo, Y. Shi, P. Liu, et al., “Dual Improvement in Sensitivity and Humidity Tolerance of a NO_2_ Sensor Based on 3-Aminopropyltriethoxysilane Self-Assembled Monolayer-Functionalized SnSe_2_ for Explosive Photolysis Gas Detection,” *ACS Applied Materials & Interfaces* *15*, no. 23 (2023): 28358–28369, https://doi.org/10.1021/acsami.3c02782.

3. K. Rathi and K. Pal, “Fabrication of MoSe_2_–Graphene Hybrid Nanoflakes for Toxic Gas Sensor with Tunable Sensitivity,” *Advanced Materials Interfaces* *7*, no. 12 (2020): 2000140, https://doi.org/10.1002/admi.202000140.

4. C. Liu, X. Chen, H. Luo, et al., “Highly Sensitive and Recoverable Room-Temperature NO_2_ Gas Detection Realized by 2D/0D MoS_2_/ZnS Heterostructures with Synergistic Effects,” *Sensors and Actuators B: Chemical* *347* (2021): 130608, https://doi.org/10.1016/j.snb.2021.130608.

5. Y. Han, D. Huang, Y. Ma, et al., “Design of Hetero-Nanostructures on MoS_2_ Nanosheets to Boost NO_2_ Room-Temperature Sensing,” *ACS Applied Materials & Interfaces* *10*, no. 26 (2018): 22640–22649, https://doi.org/10.1021/acsami.8b05811.

6. S. Kumar, A. Betal, A. G. Chakkar, et al., “MoS_2_–PdS_2_ Heterostructure for NO_2_ Sensing under Ambient Conditions: An Experimental and Computational Study,” *ACS Applied Materials & Interfaces* *17*, no. 50 (2025): 68157–68168, https://doi.org/10.1021/acsami.5c18308.

7. L. Zhang, Y. Liang, L. Yu, H. Wang, and M. Yin, “Activating and Modifying the Basal Planes of MoS_2_ for Superior NO_2_ Sensing at Room Temperature,” *Sensors and Actuators B: Chemical* *359* (2022): 131539, https://doi.org/10.1016/j.snb.2022.131539.

8. A. N. Kumar, and K. Pal, “Amine-Functionalized Stable Nb_2_CT_x_ MXene toward Room Temperature Ultrasensitive NO_2_ Gas Sensor,” *Materials Advances* *3*, no. 12 (2022): 5151–5162, https://doi.org/10.1039/D2MA00301E.

9. K. Zhao, X. Chang, J. Zhang, F. Yuan, and X. Liu, “Electronic Modulation of MoS_2_ Nanosheets by N-Doping for Highly Sensitive NO_2_ Detection at Room Temperature,” *ACS Sensors* *9*, no. 1 (2024): 388–397, https://doi.org/10.1021/acssensors.3c02148.

10. Q. Zhao, D. Sun, S. Wang, et al., “Enhanced Blocking Effect: A New Strategy to Improve the NO_2_ Sensing Performance of Ti_3_C_2_T_x_ by γ-Poly (L-Glutamic Acid) Modification,” *ACS Sensors* *6*, no. 8 (2021): 2858–2867, https://doi.org/10.1021/acssensors.1c00132.

11. S. Hersberger, M. Pereira Martins, S. Fassbind, and A. T. Güntner, “Selective, Stable and Humidity-Robust NO_2_ Sensing at Room Temperature with Porous WS_2_ Films,” *ACS Sensors* *10*, no. 11 (2025): 8596–8605, https://doi.org/10.1021/acssensors.5c02414.

12. Q. Zhao, W. Zhou, M. Zhang, et al., “Edge-Enriched Mo_2_TiC_2_T_x_/MoS_2_ Heterostructure with Coupling Interface for Selective NO_2_ Monitoring,” *Advanced Functional Materials* *32*, no. 39 (2022): 2203528, https://doi.org/10.1002/adfm.202203528.

13. P. Bharathi, S. Harish, M. Shimomura, et al., “Conductometric NO_2_ Gas Sensor Based on Co-Incorporated MoS_2_ Nanosheets for Room Temperature Applications,” *Sensors and Actuators B: Chemical* *360* (2022): 131600, https://doi.org/10.1016/j.snb.2022.131600.
